# Photolithography-Induced Doping and Interface Modulation for High-Performance Monolayer WSe_2_ P-Type Transistors

**DOI:** 10.1021/acs.nanolett.4c06407

**Published:** 2025-02-21

**Authors:** Yu-Tung Lin, Yu-Wei Hsu, Zih-Yun Fong, Ming-Yu Shen, Ching-Hao Hsu, Shu-Jui Chang, Ying-Zhan Chiu, Shao-Heng Chen, Nien-En Chiang, I-Chih Ni, Tsung-En Lee, Chih-I Wu

**Affiliations:** § Graduate Institute of Photonics and Optoelectronics, National Taiwan University, Taipei 106, Taiwan; ¶ Graduate School of Advanced Technology, National Taiwan University, Taipei 106, Taiwan; † Department of Microelectronics, National Yang Ming Chiao Tung University, Hsinchu 300, Taiwan; ‡ International College of Semiconductor Technology, National Yang Ming Chiao Tung University, Hsinchu 300, Taiwan

**Keywords:** two-dimensional materials, tungsten diselenide, two-step lithography, field-effect transistor, interface modulation, contact resistance reduction, interface analysis, photoemission spectroscopy

## Abstract

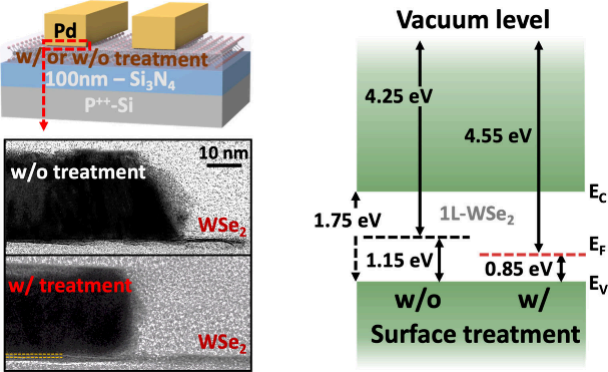

To mitigate Fermi-level pinning (FLP) at the contact of two-dimensional (2D) transition metal dichalcogenides and enhance their hole carrier concentration, a 1.8 nm-thick p-doping layer is formed via photolithography. This surface treatment significantly reduces the contact resistance (*R*_C_) to ∼4.8 kΩ·um in monolayer (1L) WSe_2_ p-type field-effect transistors (p-FETs) and increases hole carrier concentration by 1.4 times, resulting in a field-effect mobility of ∼75 cm/V·s. After subsequent helium ion-beam lithography, the Fermi level can still be modulated from 4.25 to 4.55 eV due to the ultrathin buffer layer. This approach enables high-performance p-FETs with 1L-WSe_2_ channels, achieving a maximum on-state current density of 420 μA/μm at a *V*_D_ of −1 V and ultralow *R*_C_ of ∼0.8 kΩ·um by the combination of the MoO_*x*_ encapsulation for additional p-doping. These results demonstrate that 1L-WSe_2_ p-FETs can attain performance comparable to 2D n-FETs, paving the way for high-performance complementary metal-oxide semiconductor transistors with 2D channels.

To push semiconductor scaling limits and sustain Moore’s law, ultrathin two-dimensional (2D) transition metal dichalcogenides (TMDs) have emerged as promising candidates for next-generation electronic applications.^1−3^ These materials offer atomically smooth, bonding-free surfaces that minimize surface scattering, enabling remarkable electrical performance at the monolayer (1L) level.^4^ Moreover, their strong electrostatic control provides robust immunity to short-channel effects, positioning TMDs as ideal channels for future scaled devices.^5,6^ Employing TMDs in complementary field-effect transistors (CFETs) facilitates extreme vertical scaling. Given the need for both high-performance p-FETs and n-FETs in CFET integration, molybdenum disulfide n-FETs have demonstrated high current density and low contact resistance, particularly using bismuth or antimony as contact metals.^7−10^ Among TMDs for p-type channels, tungsten diselenide (WSe_2_) stands out due to its tendency for p-type behavior, stable conduction, and high hole mobility of ∼100–300 (cm^2^/V·s).^11−13^

However, Fermi-level pinning (FLP) caused by metal-induced gap states (MIGS) and disorder-induced gap states (DIGS) results in the Fermi level being fixed at a position that poorly aligns with the desired band edge.^14−18^ This misalignment leads to high contact resistance (*R*_C_) as the injection of holes from the metal into the TMD channel becomes less efficient. Therefore, methods to alleviate FLP are essential for improving device performance. Several strategies have been proposed to reduce FLP and *R*_C_, including the use of semimetallic contacts or the insertion of interlayers between the TMD–metal interface.^19−21^ Additionally, intentional doping at the contact region can enhance band bending, which narrows the Schottky barrier width (SBW) and increases total current flow.^22−25^

This work presents a novel surface treatment method that employs a two-step lithography technique to create a p-doping layer, aiming to relieve the FLP issue and tune the effective SBH at the WSe_2_ contact interfaces. Various interface characteristics have been investigated to confirm the underlying mechanism of this surface treatment: (1) low-temperature Raman and photoluminescence (PL) spectroscopy to assess the material properties following doping layer application and subsequent helium ion-beam lithography (HIBL); (2) X-ray photoelectron spectroscopy (XPS) to analyze the electron configuration of 1L-WSe_2_ after surface treatment; and (3) ultraviolet photoelectron spectroscopy (UPS) to characterize the Fermi level of surface-treated 1L-WSe_2_ after contact with a high work function metal.

The schematic in Figure 1a illustrates the fabrication flow of 1L-WSe_2_ p-FETs, highlighting the two-step lithography technique, consistinf of photolithography and HIBL employed to modify 1L-WSe_2_ surfaces and fabricate scaled devices. This surface treatment was specifically applied to address FLP at the metal–2D interfaces while simultaneously enhancing hole carrier concentration through the formation of a thin layer on WSe_2_. To study the impacts of surface treatment on device performance, untreated devices were fabricated by the same process except photolithography. The transfer characteristics of treated and untreated devices with a channel length (*L*_CH_) of 2 μm, measured at *V*_D_ = −1 V, are presented in Figure 1b. The red and black curves correspond to the treated and untreated devices, respectively. Devices with surface treatment exhibit a significant enhancement in drain current (*I*_D_) across the same gate voltage range. Additionally, a positive shift in threshold voltage (*V*_TH_) is attributed to the doping effect introduced by the surface treatment. To further examine the carrier injection properties at the WSe_2_ contact region, the output characteristics of both treated and untreated devices are shown in Figure 1c. The surface-treated devices demonstrate a high maximum *I*_D_ of 52 μA/μm at *V*_D_ = −2 V, approximately five times higher than that of untreated devices. Moreover, untreated devices exhibit nonlinear curves, indicative of poor carrier injection and high *R*_C_, whereas surface-treated WSe_2_ p-FETs exhibit linear behavior, enabled by a lower effective hole Schottky barrier and efficient direct tunneling through the ultrathin p-doping layer.

**Figure 1:**
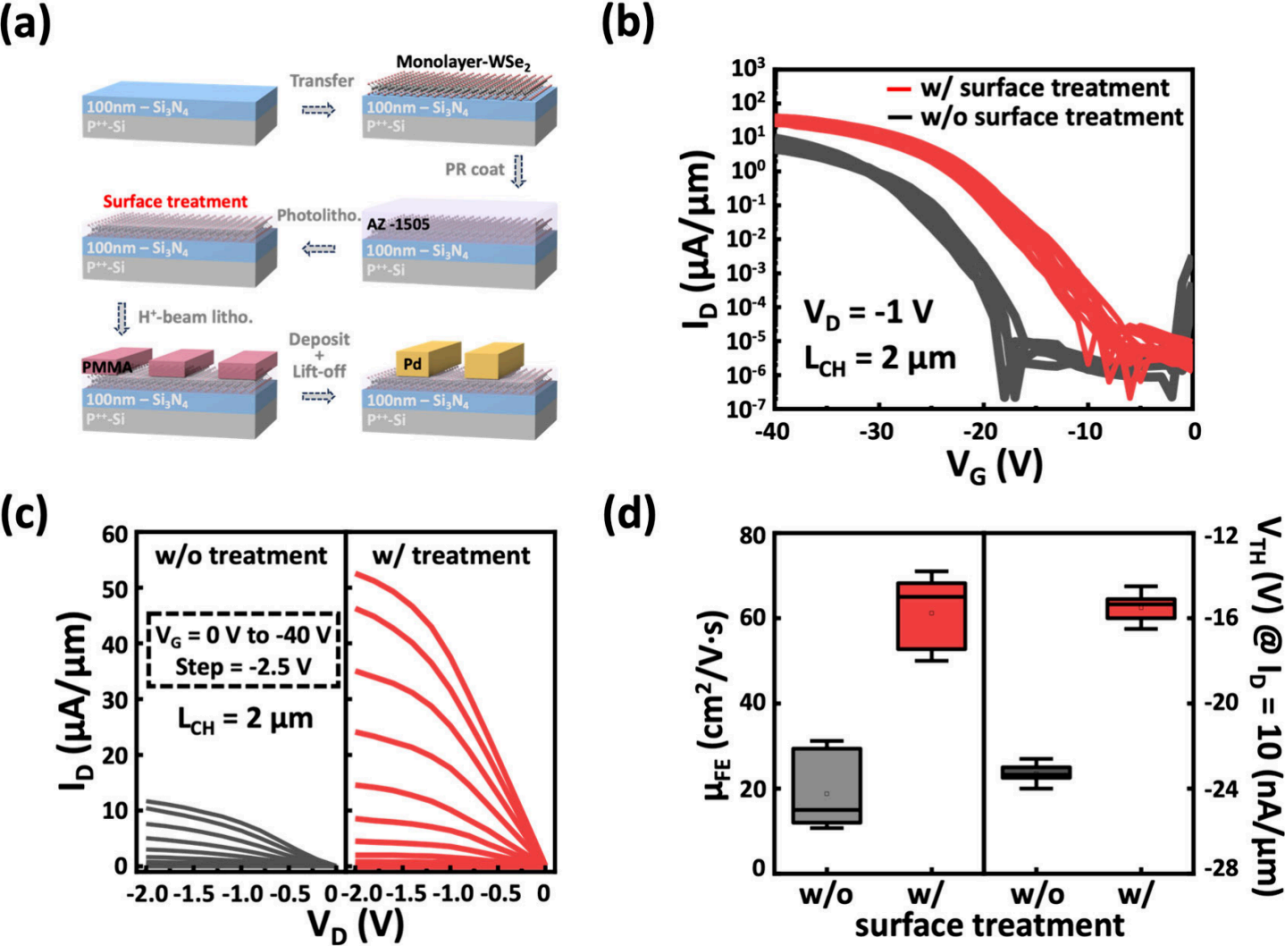
Statistical electrical characteristics of 1L-WSe_2_ p-FETs on a Si/100 nm-Si_3_N_4_ substrate with and without surface treatment. Device channel length and width are 2 and 20 μm, respectively. (a) Schematic illustration and process flow for fabricating surface-treated devices. (b) Comparison of transfer characteristics and (c) output characteristics between devices with and without surface treatment. (d) Summary of field-effect mobility and threshold voltage at a fixed current density from data in panel b.

Figure 1d summarizes the field-effect mobility (μ_FE_) and *V*_TH_ for both treated and untreated devices, extracted from the transfer characteristics in Figure 1b. μ_FE_ was enhanced 3 times higher from an average value of 20 to 60 cm^2^/V·s, indicating an interface with lower *R*_C_ and less scattering centers. Furthermore, the positive *V*_TH_ shifts from −23 to −15 V at a fixed *I*_D_ of 10 nA/μm, which implies the increase in the maximum hole carrier concentration from 7 × 10^12^ to 1 × 10^13^ cm^–2^ under the same voltage sweep. Moreover, this positive *V*_TH_ shift modulated by the surface treatment is beneficial to matching the *V*_TH_ between p-FET and n-FET composing the CMOS inverter. Furthermore, we applied the same surface treatment process to MoS_2_ with Au contacts, which also exhibited a slight p-doping effect, further demonstrating the compatibility of this approach (shown in Figure S1).

To study the effects of surface treatment on the 1L-WSe_2_ film, atomic force microscopy (AFM) was used to analyze the surface morphology, as shown in Figure 2a. While the treated sample exhibits a slightly rougher surface compared to the untreated one, likely due to the formation of polymer layers, the low variability in *V*_TH_ observed in both treated and untreated devices, in Figure 1b, suggests that the doping position in the layer is located nearly at the interface of 1L-WSe_2_.

**Figure 2:**
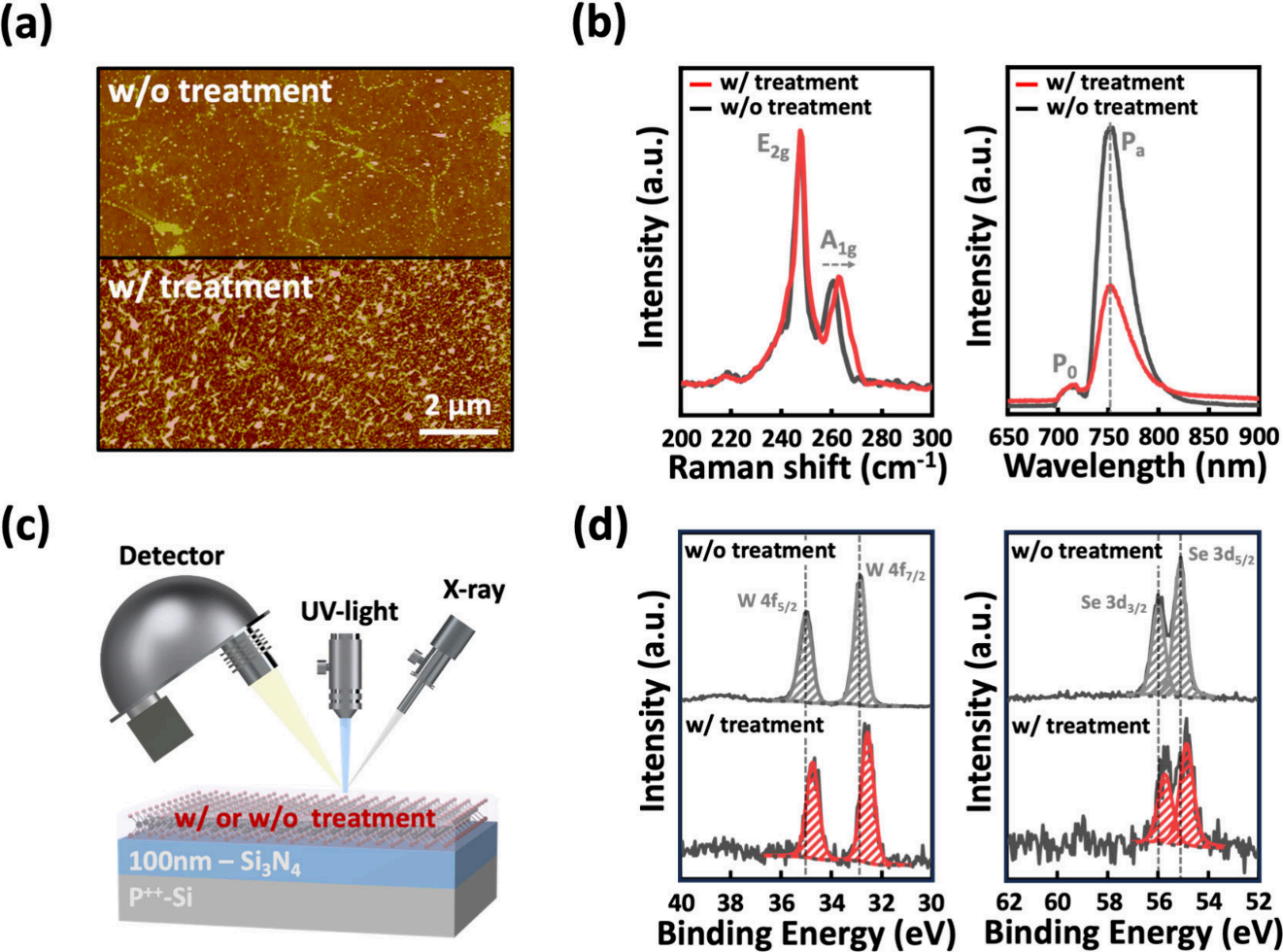
Material and surface characteristic of the 1L-WSe_2_ film. (a) AFM images and (b) low-temperature Raman/PL spectra of 1L-WSe_2_, comparing treated and untreated samples. (c) Schematic image of the XPS measurement setup. (d) XPS spectra of W *4f* and Se *3d* core levels for 1L-WSe_2_, illustrating peak differences between treated and untreated surfaces.

To further correlate the doping effects with the electrical characteristics, low-temperature Raman and PL spectra were measured, as shown in Figure 2b. At 10 K, the thermal expansion and vibrations are significantly suppressed, allowing the intrinsic properties of 1L-WSe_2_ to be probed with minimal thermal interference.^26−28^ Raman analysis reveals that the E_2g_ peak remains unchanged after treatment, indicating negligible strain effects on the 1L-WSe_2_ film capped by the polymer layer. In contrast, the A_1g_ peak exhibits a pronounced blue shift, which is attributed to the effective p-doping effect introduced by the capping layer.^29−31^ This shift reflects the increased hole size *n*_2D_, which modifies electron–phonon coupling and influences the out-of-plane vibrational mode. Next, the PL spectrum reveals two prominent features: a band-edge exciton emission (P_0_) of ∼710 nm, corresponding to the direct bandgap energy of 1.74 eV, and an additional emission peak (P_a_) at ∼750 nm, associated with a shallow defect level within the bandgap.^32^ The emission at 1.64 eV is indicative of defect states, likely introduced during the transfer process or arising from intrinsic material quality. After surface treatment, the intensity of the P_a_ peak decreases due to the p-doping effect. This doping introduces excess holes that suppress radiative recombination, thereby reducing PL intensity.^33−35^

To examine the chemical composition and bonding states of the W–Se bonds in treated and untreated 1L-WSe_2_, XPS was performed as illustrated in Figure 2c. The XPS spectra of the W *4f* and Se *3d* core levels are shown in Figure 2d. The W *4f* spectrum exhibits two primary peaks at 35.05 and 32.85 eV, corresponding to the W *4f*_*5/2*_ and W *4f*_*7/2*_ states, respectively. Similarly, the Se *3d* spectrum shows peaks at 55.95 and 55.10 eV, assigned to the Se *3d*_*3/2*_ and Se *3d*_*5/2*_ states, characteristic of the W–Se bonds in intrinsic WSe_2_.^36^ After surface treatment, both the W *4f* and Se *3d* spectra exhibit a shift to lower binding energies by approximately 0.3 eV, confirming the p-doping effect introduced by the treatment. This shift is likely attributed to the residual polymer layers formed on the WSe_2_ surface during the photolithography process. The doping layer thickness is discussed later. As photoresists such as AZ1505 undergo UV exposure and development, they can act as Lewis acids, attracting electrons from 1L-WSe_2_. This interaction modifies the surface chemistry of 1L-WSe_2_, enhancing the hole carrier concentration.^37^

To further examine the impacts of surface treatment on the contact properties and device performance, devices with *L*_CH_ scaled down to sub-100 nm were investigated. Figure 3a shows the transfer characteristics of more than 100 devices with an *L*_CH_ of 100 nm at a *V*_D_ of −1 V, comparing treated and untreated devices. Consistent with the trends observed for an *L*_CH_ of 2 μm in Figure 1b, the treated devices (red curves) exhibit significantly higher *I*_D_ compared to untreated devices (black curves). The difference in *I*_D_ becomes more pronounced at shorter *L*_CH_, which is attributed to the contact-dominated transport behavior in these devices. As *R*_C_ constitutes a larger portion of the total resistance (*R*_total_) at shorter *L*_CH_, the treated devices demonstrate a substantial reduction in *R*_C_ due to the improved contact interface. This highlights the effectiveness of the treatment in mitigating *R*_C_, particularly for the aggressively scaled devices. Figure 3b summarizes the extracted on-state *I*_D_ and *R*_C_ values using the Y-function method at a fixed carrier concentration of ∼7 × 10^12^ cm^–2^. Surface-treated devices demonstrate a remarkable enhancement in current density, achieving values approximately seven times higher than those of untreated devices. Moreover, the *R*_C_ of treated devices is reduced by 1 order of magnitude. These improvements underscore the critical role of surface treatment in optimizing contact properties, particularly after HIBL and Pd deposition. While the surface treatment approach has significantly improved overall mobility and reduced contact resistance, the subthreshold swing (SS) remains suboptimal due to the use of a 100 nm Si_3_N_4_ substrate in bottom-gated devices, as shown in Figure S2. Achieving an ideal SS is crucial for low-power consumption in advanced logic applications. To address this, fabricating 1L-WSe_2_ p-FETs on thinner high-*k* dielectrics and adopting a top-gate or dual-gate configuration can enhance electrostatic control by reducing the effective oxide thickness, thereby improving the SS performance.

**Figure 3:**
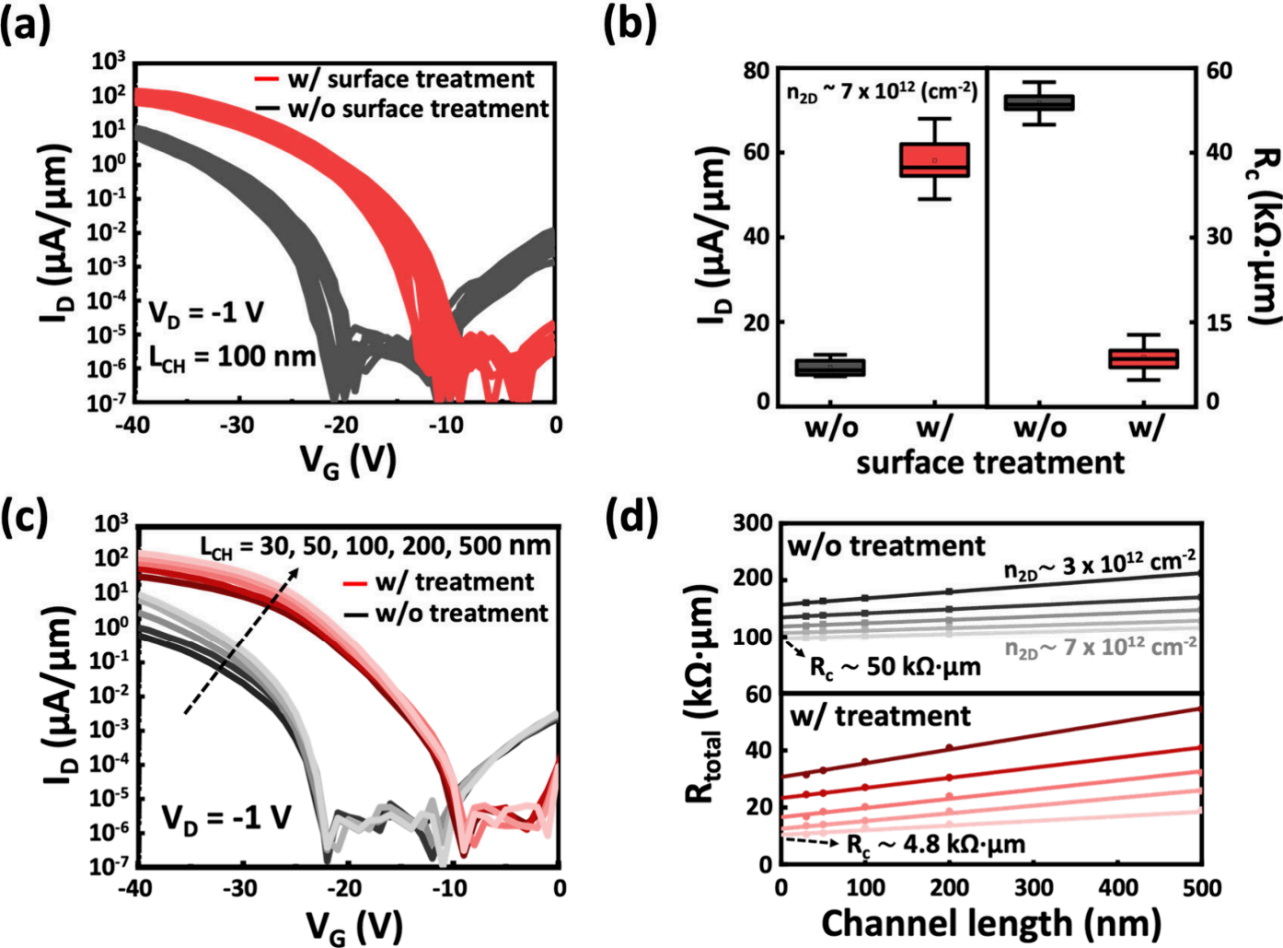
Statistical comparison of electrical characteristics for devices with and without surface treatment. (a) Transfer characteristics measured at *V*_D_ = −1 V for 100 devices with a channel length of 100 nm. (b) On-state current and contact resistance extracted using the Y-function method at fixed carrier concentration, based on data from Figure 3a. (c) Transfer characteristics for devices with channel lengths ranging from 30 to 500 nm at *V*_D_ = −1 V. (d) Contact resistance extraction using the TLM method.

To further evaluate the device performance, transfer curves for *L*_CH_ ranging from 500 to 30 nm are measured at *V*_D_ = −1 V, shown in Figure 3c. Both treated and untreated devices exhibit consistent *V*_TH_ positions across various *L*_CH_, indicating a uniform material quality and stable fabrication processes. The validity of the *R*_C_ extraction was further confirmed using the transfer length method (TLM), as illustrated in Figure 3d. Treated devices consistently exhibit lower *R*_C_ compared to untreated devices across the entire channel length range, with carrier concentrations (*n*_2D_) varying from 3 × 10^12^ to 6 × 10^12^ cm^–2^. At an *n*_2D_ of 6 × 10^12^ cm^–2^, the minimum *R*_C_ is significantly reduced from 50 to 4.8 kΩ·μm with the application of surface treatment, underscoring improved charge injection and transport efficiency. The substantial reduction in *R*_C_ reveals the critical role of surface treatment in achieving a superior 1L-WSe_2_ p-FETs performance. Additionally, as shown in Figure S3, the devices demonstrate excellent stability, supporting the robustness and scalability of this approach.

To elucidate the physical mechanism underlying the reduced *R*_C_ and increased hole carrier concentration in the contact region of 1L-WSe_2_ after surface treatment, a detailed physical analysis was conducted on both treated and untreated 1L-WSe_2_ films subjected to HIBL. Figure 4a shows the low-temperature Raman spectra of the treated and untreated samples following HIBL. The treated sample retains a noticeable blue shift in the A_1g_ peak, indicating the sustained p-doping effect. This observation aligns with the observations in Figure 2b, confirming that the doping strength from the residual polymer layers remains even after exposure to helium ion-beam bombardment.

**Figure 4:**
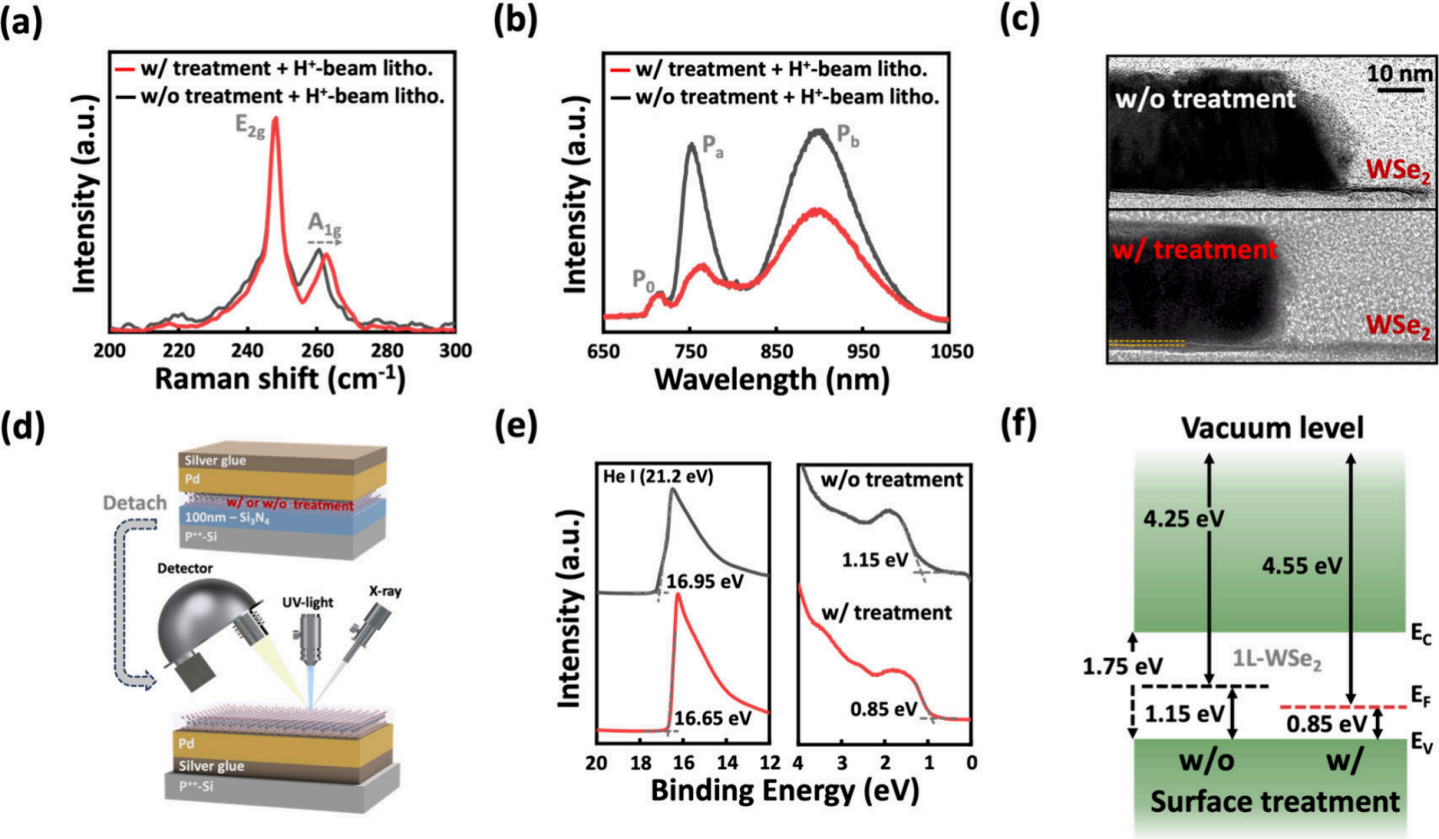
Characterization of the contact region in 1L-WSe_2_ p-FETs. (a) Raman and (b) PL spectra comparing treated and untreated 1L-WSe_2_ following helium-ion beam lithography. (c) Cross-sectional TEM images of Pd/1L-WSe_2_ interfaces with and without surface treatment. (d) Schematic of sample preparation and the measurement setup for UPS analysis. (e) Onset and cutoff regions for UPS spectra in treated and untreated samples. (f) Band alignment diagram at the Pd/1L-WSe_2_ interface with these two cases based on the data of Figure 4e.

Next, the low-temperature PL spectra in Figure 4b reveal the presence of an additional emission peak (P_b_) at ∼900 nm (corresponding to an energy position of ∼1.38 eV), which is attributed to defect states induced by ion-beam bombardment.^38,39^ In comparison, the P_a_ peak observed in untreated samples originates from shallow defect states within the bandgap, as discussed in Figure 2b, while P_b_ corresponds to deeper defect levels formed due to ion-beam damage. Ion-beam lithography is known to generate defects in 1L-WSe_2_, thereby degrading the material quality in the contact region. However, the intensity of P_b_ is significantly reduced for the treated one. These findings suggest that the p-doping layer introduced during surface treatment serves as a protective barrier, mitigating the formation of additional defects in the contact region during HIBL. This highlights the critical role of surface treatment in reserving low *R*_C_ values under ion-beam processing.

The contact interface was further examined by using cross-sectional transmission electron microscopy (TEM) images of the palladium (Pd)/1L-WSe_2_ contact, with and without surface treatment, as shown in Figure 4c. In untreated devices, the interface displays significant intermixing between the metal contact and the WSe_2_, indicative of defect formation during the Pd metallization process. In contrast, the treated one displays a distinct ultrathin interlayer between the metal and WSe_2_. The presence of such interlayers at the TMD–metal interface is a well-recognized approach for mitigating FLP caused by lithography and metal deposition processes.^40,41^ The thickness of this interlayer estimated to be ∼1.8–2.0 nm using the XPS attenuation model is shown in Figure S4, while additional TEM images providing a clear view of the metal contact and WSe_2_ interface are shown in Figure S5.

To clarify the band alignment at the contact region of treated and untreated 1L-WSe_2_, UPS measurements were conducted to determine the effective work function of the Pd/1L-WSe_2_ interface. The schematic of the UPS measurement setup is shown in Figure 4d, with the corresponding UPS spectra shown in Figure 4e. The valence band maximum (VBM) position of WSe_2_ in contact with Pd was derived from the slope of the onset region in the spectra, using the Fermi level (*E*_F_) as the zero-energy reference. The extracted *E*_F_-VBM values are 1.15 and 0.85 eV for the untreated and treated samples, respectively. On the left side of Figure 4e, the secondary electron cutoff spectra were used to determine the effective work function of Pd contacts, calculated as 4.25 eV for the untreated sample and 4.55 eV for the treated sample at the WSe_2_ interface. The energy band alignment for the contact configurations on 1L-WSe_2_ is summarized in Figure 4f, based on the XPS and UPS analysis in Figure 4e. Surface treatment increased the effective work function by 0.3 eV, positioning it closer to the VBM. This shift is consistent with the XPS results shown in Figure 2d, further proving the p-doping effect introduced by the treatment. These adjustments in band alignment result in a significant reduction in the effective Schottky barrier, facilitating an improved hole injection at the contact interface. The correlation between these findings and the enhanced device performance underscores the critical role of the surface treatment in optimizing 1L-WSe_2_ p-FETs.

To further enhance the performance of 1L-WSe_2_ p-FETs, an additional doping layer was deposited on 1L-WSe_2_ to achieve a higher hole carrier concentration. Molybdenum oxide (MoO_*x*_), deposited by thermal evaporation, was selected as the capping material due to its high electron affinity (5.2–6.9 eV).^42−44^ The band alignment before and after MoO_*x*_ encapsulation is schematically illustrated in Figure 5a. The MoO_*x*_ layer induces a significant realignment of *E*_F_, further enhancing the hole doping level in 1L-WSe_2_ via surface charge transfer. The transfer characteristics in Figure 5b show additional improvement in both treated and untreated devices after MoO_*x*_ encapsulation. Notably, treated devices with MoO_*x*_ encapsulation achieve an *I*_D_ ∼ 420 μA/μm at *V*_D_ = −1 V, accompanied by an on/off current ratio exceeding 10^8^. Similarly, the output characteristics in Figure 5c reveal excellent linearity and higher *I*_D_ values, reaching 750 μA/μm at *V*_D_ = −2 V. These results signify a significant reduction in *R*_C_ and improved carrier transport properties.

**Figure 5:**
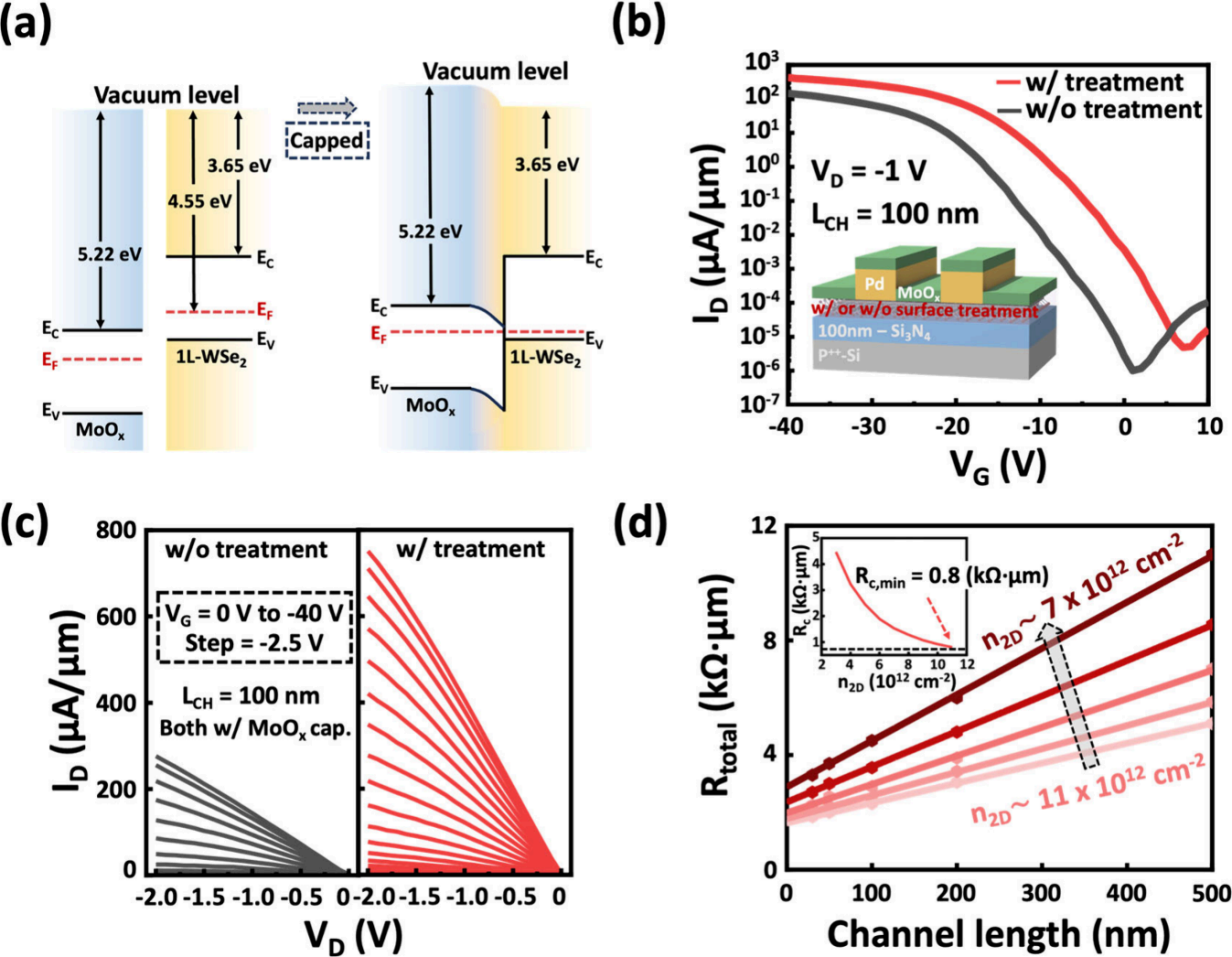
Device characteristics with optimized MoO_*x*_ encapsulation. (a) Schematic illustration of band alignment for 1L-WSe_2_ before and after MoO_*x*_ capping. (b) Transfer characteristics and (c) output characteristics comparison between devices with and without surface treatment after MoO_*x*_ capping. (d) Contact resistance extraction for surface-treated devices after MoO_*x*_ capping, using the TLM method. Inset: Contact resistance versus carrier concentration, demonstrating a minimum contact resistance of ∼0.8 kΩ·μm.

To quantitatively analyze the contact properties, TLM measurements were conducted, shown in Figure 5d, with detailed extraction in Figure S6. The *n*_2D_ in treated devices after MoO_*x*_ encapsulation increases to 7 × 10^12^–1.1 × 10^13^ cm^–2^, attributed to the strong doping effect of MoO_*x*_. Remarkably, an ultralow *R*_C_ ∼ 0.8 kΩ·μm is achieved at a carrier concentration of ∼1.1 × 10^13^ cm^–2^, representing one of the lowest reported values for 1L-WSe_2_ p-FETs with an on/off current ratio of ∼8 orders of magnitude (see Figure S7 and Table S2).

In conclusion, we successfully demonstrate a scalable strategy to reduce the effective Schottky barrier at the contact interface of 1L-WSe_2_ p-FETs using an ultrathin p-doping layer introduced via photolithography. The surface treatment reduces *R*_C_ ∼ 4.8 kΩ·μm, while MoO_*x*_ encapsulation further enables ultralow *R*_C_ ∼ 0.8 kΩ·μm, a high on-state current of 420 μA/μm, and an on/off ratio exceeding 10^8^. The physical mechanisms underlying the reduced *R*_C_ and higher μ_FE_ are attributed to higher hole carrier concentrations and reduced defect density at the contact/1L-WSe_2_ interface, both before and after HIBL. These results highlight a promising pathway for high-performance 2D p-MOS devices and their integration into future CMOS technologies with 2D monolayer channels.

## Methods

### Fabrication of the 1L-WSe_2_ p-FETs with Surface Treatment

The CVD-grown 1L-WSe_2_ film was precisely transferred onto a Si/100 nm-Si_3_N_4_ substrate. To remove residues from the transfer process, the substrate was immersed in acetone for 24 h. This was followed by thermal annealing at 200 °C under an Ar flow for 45 min to ensure complete residue removal and proper adhesion between the WSe_2_ film and the substrate. Subsequently, the AZ1505 photoresist was spin-coated onto the WSe_2_/Si_3_N_4_ samples, with a soft-baking step at 90 °C for 90 s. A photolithography process using an μMLA maskless aligner (manufactured by Heidelberg Instruments) with a wavelength of 390 nm is employed. The lithography process is optimized by adjusting the exposure dose, defocus, and development time. The AZ1505 photoresist, upon exposure, is converted into a photoacid. When immersed in the development solution (MIF-319), the photoacid undergoes an acid–base neutralization reaction. The photoresist that is not converted into photoacid remains on the surface. This step is performed to create a polymer doping layer on the surface of the 1L-WSe_2_, referred to as the surface treatment in this study. Following the surface treatment, source and drain regions were defined using HIBL, and a 25 nm Pd layer was deposited to form the contact electrodes. The source/drain contacts were completed by using a standard metal lift-off process. Finally, the active device area was defined by O_2_ plasma etching, completing the fabrication process.

### Sample Preparation for UPS Measurement

A 25 nm Pd film was deposited onto the 1L-WSe_2_ film transferred onto a Si/100 nm-Si_3_N_4_ substrate. To minimize surface charging during measurements, silver glue was applied to the Pd film, which was subsequently bonded to the heavily doped silicon substrate. Before analysis, the Si/100 nm-Si_3_N_4_ substrate was removed, and the sample was immediately transferred to the characterization chamber to preserve surface cleanliness. Notably, the bonding procedure using silver paste was performed entirely in a glovebox under controlled conditions to eliminate moisture interference and maintain a stable atmosphere.

### Optical Measurements

Low-temperature PL and Raman measurements were conducted by using a custom-built optical microscope in a backscattering configuration. A 532 nm solid-state laser served as the excitation source and was focused onto the sample with a 100× objective lens. Signals were collected through the same objective lens, analyzed using a 0.75 m monochromator, and detected by a liquid-nitrogen-cooled CCD camera. The laser beam was focused to a spot size of approximately 1 μm on the sample. For additional optical measurements, a supercontinuum white-light laser (λ: 500–2400 nm) was dispersed by a monochromator and focused onto the sample with the same 100× objective lens. The transmitted light was collected from the rear side using another 100× objective lens and detected by a silicon diode (λ: 500–900 nm) or a germanium diode (λ: 700–1400 nm).

### PES Measurements

XPS was performed using a ULVAC PHI 5000 Versa Probe III to analyze changes in the chemical state. The measurements were conducted with a monochromatic Al Kα source (*h*ν = 1486.6 eV) at 25 W power, a 15 kV accelerating voltage, and a 100 μm spot size. High-resolution spectra were acquired with a pass energy of 27.00 eV and a step size of 0.05 eV. To further investigate the electronic band structure, UPS was carried out on the same instrument using a He I excitation line (*h*ν = 21.2 eV).

## Supporting Information Available

Transfer characteristics of 1L-MoS_2_ with and without surface treatment; summary of subthreshold swing at a fixed current density; transfer characteristics of monolayer WSe_2_ p-FETs with surface treatment devices’ stability assessed after 7 and 30 days; thickness estimation of a polymer doping layer induced by surface treatment determined using the XPS attenuation model; transfer characteristics of monolayer WSe_2_ p-FETs with surface treatment and MoO_*x*_ encapsulation measured for channel lengths ranging from 30 to 500 nm; benchmark of maximum *I*_D_ versus on/off ratio; performance metrics for monolayer and multilayer WSe_2_ field-effect transistors (PDF)
