# Supplementary material for: Photolithography-Induced Doping and Interface Modulation for High-Performance Monolayer WSe2 P-Type Transistors
Source: Nano Lett. 2025 Feb 21;25(9):3571–8. doi: 10.1021/acs.nanolett.4c06407 (PMC11887443; doi:10.1021/acs.nanolett.4c06407)
Supplement: Supplementary file 1 — nl4c06407_si_001.pdf [file nl4c06407_si_001.pdf]

Supporting information

Photolithography-Induced Doping and Interface Modulation for High-Performance Monolayer WSe_2 P-Type Transistors

Yu-Tung Lin,[§,∥] Yu-Wei Hsu,[¶,∥] Zih-Yun Fong,[§] Ming-Yu Shen,[§] Ching-Hao Hsu,[§] Shu-Jui Chang,[‡] Ying-Zhan Chiu,[¶] Shao-Heng Chen,[§] Nien-En Chiang,[¶] I-Chih Ni,[§] Tsung-En Lee,[†] and Chih-I Wu*[§,¶]*

[§]Graduate Institute of Photonics and Optoelectronics, National Taiwan University, Taipei 106, Taiwan

[¶]Graduate School of Advanced Technology, National Taiwan University, Taipei 106, Taiwan

[†]Department of Microelectronics, National Yang Ming Chiao Tung University, Hsinchu 300, Taiwan

[‡]International College of Semiconductor Technology, National Yang Ming Chiao Tung University, Hsinchu 300, Taiwan

Corresponding Authors

Chih-I Wu — Email: chihiwu@ntu.edu.tw

Tsung-En Lee — Email: telee@nycu.edu.tw

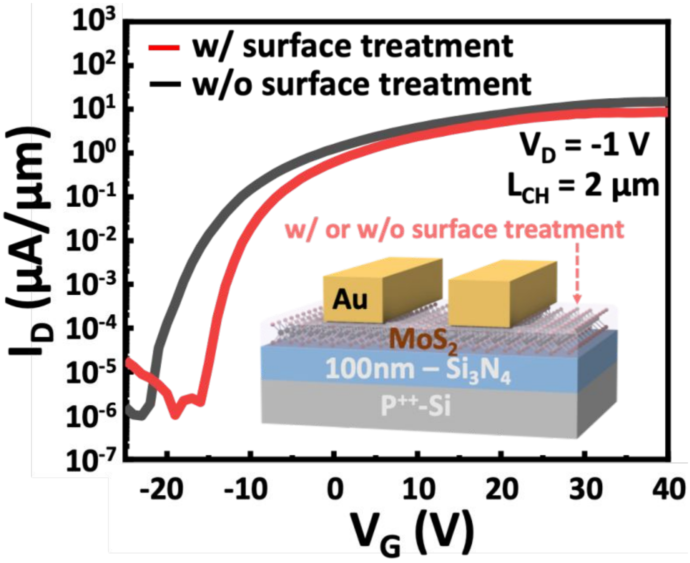

Figure S1. Transfer characteristics of 1L-MoS_2 n-FETs on Si/100nm-Si_3N_4 substrate with and without surface treatment

The transfer characteristics of MoS_2 devices, with and without surface treatment, exhibit a slight p-doping effect after treatment. However, this effect is less pronounced compared to WSe_2 devices, primarily due to the intrinsic electronic differences between the two materials. MoS_2 has a larger bandgap[1,2] and a lower valence band maximum (VBM)[3-5] than WSe_2, making hole carrier injection from the Fermi level to the VBM more challenging.

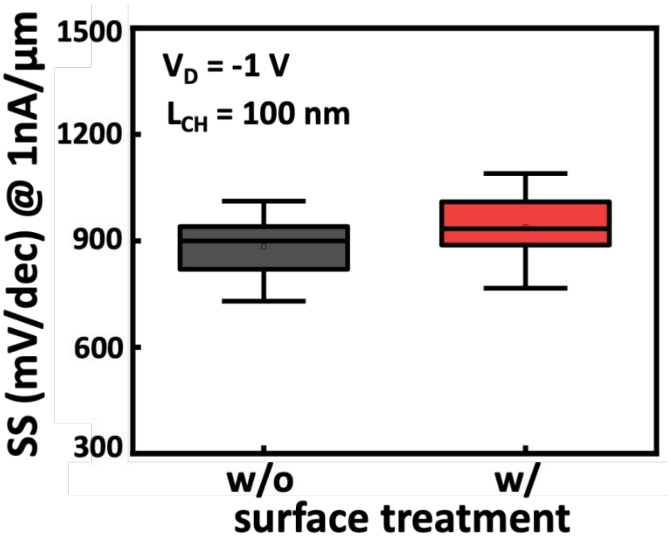

Figure S2. Summary of subthreshold swing at a fixed current density, derived from the data presented in Figure 3a.

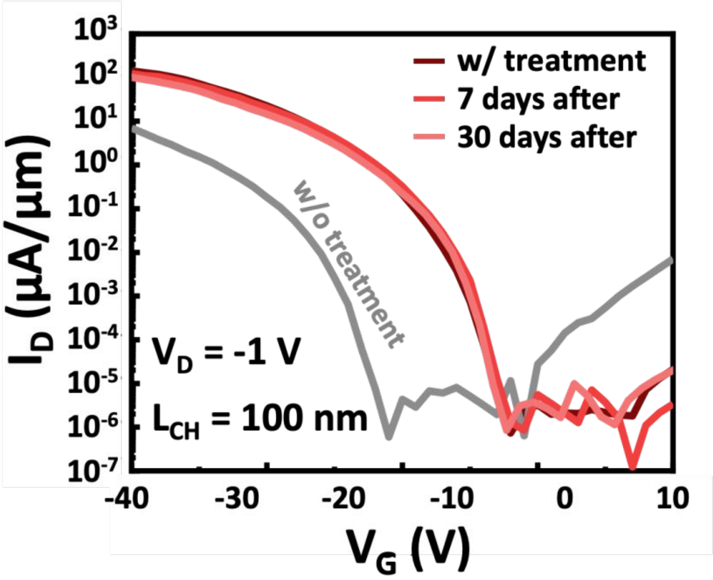

Figure S3. Transfer characteristics of 1L-WSe_2 p-FETs with and without surface treatment and device stability measurements after 7 and 30 days.

[XPS attenuation model]

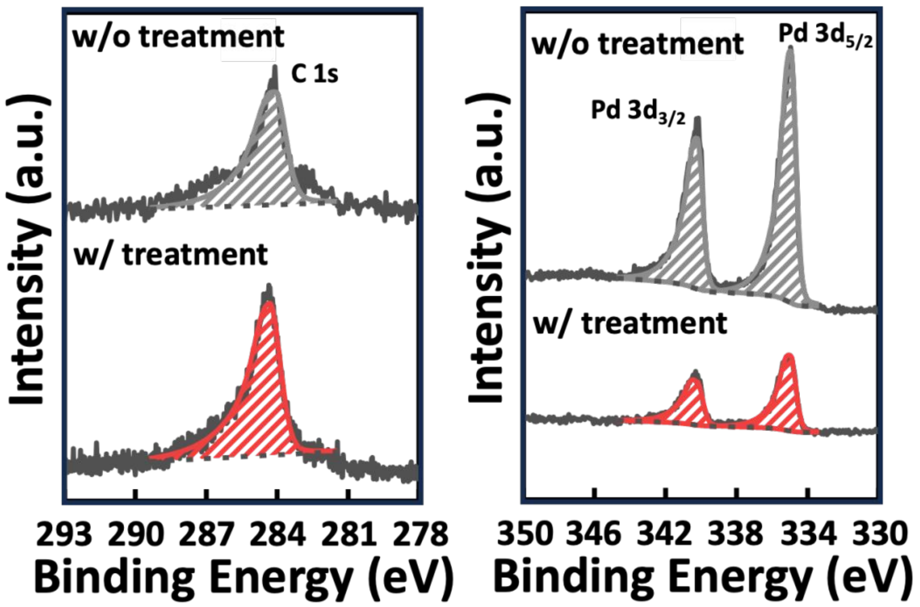

Figure S4. XPS spectra of C *1s* and Pd *3d* core levels for UPS measurement samples illustrating peak intensity differences between treated and untreated surfaces.

The XPS attenuation model is widely used to determine the thickness of thin films by analyzing the attenuation of photoelectron signals.[6-8] The thickness d can be calculated using the following formula:

$$d = \lambda \cdot \sin(\theta) \cdot \ln[(\frac{I_F}{I_S} \cdot a + 1)]$$

Here, λ represents the inelastic mean free path of the photoelectrons, θ is the photoelectron take-off angle, I_F/I_S is the intensity ratio of the overlayer to the substrate, and a is a correction factor derived from the relative sensitivity factor.

To precisely determine the thickness of the thin doping layer, the *d* value for treated samples was calculated and the corresponding value for untreated samples was subtracted. This approach corrects for polymer residues introduced during the material transfer process and measurement exposure, ensuring a more precise estimation of the doping layer thickness. As indicated in Figure S2, the calculated thickness difference between treated and untreated samples ($d_{treated}$ - $d_{untreated}$) is approximately 1.86 nm. Notably, the *Pd 3d* peak intensity of treated sample is significantly lower than that of the untreated sample, while the *C 1s* peak intensity is higher in the treated sample. These observations confirm the formation of the doping layer, corroborating the effectiveness of the surface treatment in modifying the interface characteristics.

	λ (nm)	θ	$I_F / I_S = I_{C1s} / I_{Pd3d}$	a	d (nm)
**untreated**	3.670	45°	0.1756	17.95	2.61
**treated**	3.670	45°	0.4181	17.95	3.93

Table S1. Parameters used for extracting the thickness using the XPS attenuation model formula.

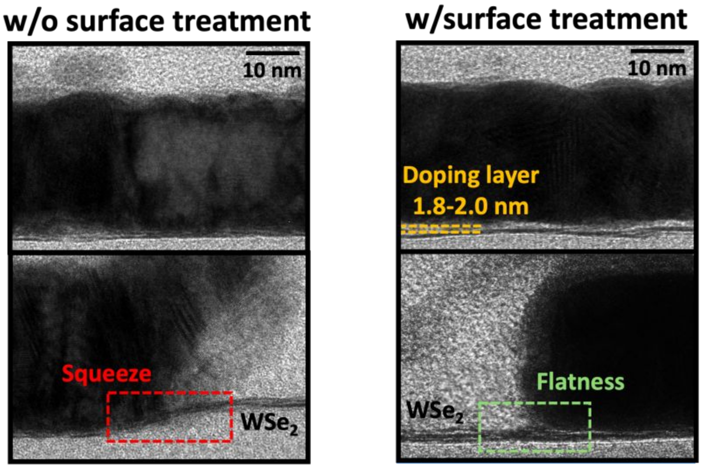

Figure S5. Additional cross-sectional TEM images of Pd/1L-WSe_2 interfaces with and without surface treatment.

An additional TEM image has been included in the supporting information to provide a clearer view of the metal contact and WSe_2 interface profile. Notably, beyond determining the doping layer thickness introduced by the surface treatment process, the TEM images reveal a significant difference at the contact edge between treated and untreated devices. Without surface treatment, WSe_2 layer is compressed and deformed by the metal contact, whereas with surface treatment, the WSe_2 maintains a smooth and uniform profile.

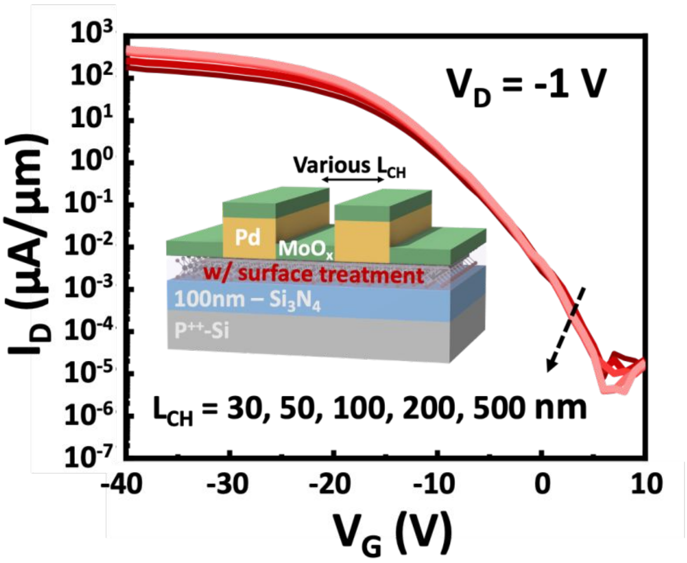

Figure S6. Transfer characteristics of 1L-WSe_2 p-FETs with surface treatment and MoO_x encapsulation measured at V_D = -1 V for channel lengths ranging from 30 to 500 nm.

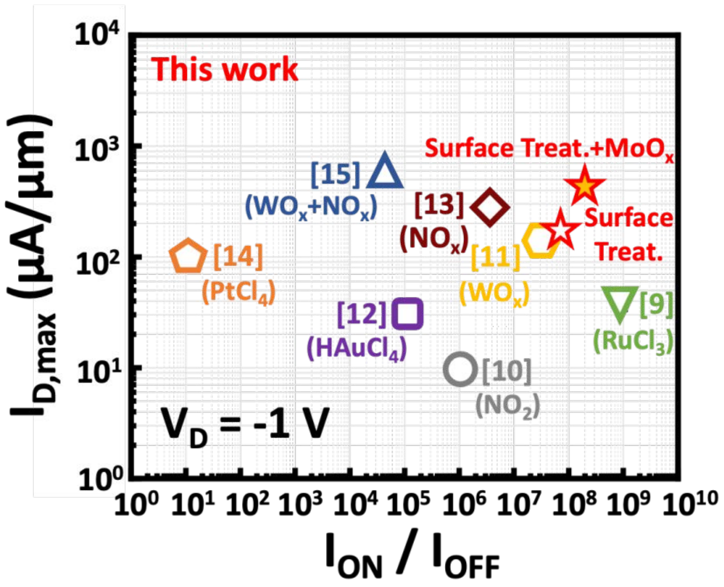

Figure S7. Benchmark of maximum I_D versus on/off ratio for monolayer WSe_2 p-FETs with existing doping techniques.

Materials	*Channel length* (nm)	$I_{D,max}$ @ V_D = -1 V (μA/μm)	*Contact resistance* (kΩ·μm)	*On/Off ratio*	*Ref.*
Exfoliated 1L-WSe_2	500	35	4.00	10^9	[9]
CVD 1L-WSe_2	65	300	0.95	10^6	[13]
Exfoliated 2L-WSe2	2000	100	0.32	<10	[14]
CVD 1L-WSe_2	55	546	0.87	10^4	[15]
Exfoliated 4L-WSe_2	400	~130	0.80	10	[16]
Exfoliated 4L-WSe_2	800	40	1.25	10^6	[17]
CVD 1L-WSe_2	100	151	0.75	10^7	[18]
CVD 1L-WSe_2	100	105	12.0	10^8	[19]
CVD 1L-WSe_2	100	420	0.80	10^8	This work

Table S2. Comparison of state-of-the-art performance metrics for monolayer and multilayer WSe_2 field-effect transistors.

REFERENCE

(1) Desai, S. B.; Seol, G.; Kang, J. S.; Fang, H.; Battaglia, C.; Kapadia, R.; Ager, J. W.; Guo, J.; Javey, A. Strain-Induced Indirect to Direct Bandgap Transition in Multilayer WSe_2. *Nano Lett.* 2014, 14 (8), 4592–4597. https://doi.org/10.1021/nl501638a.

(2) Conley, H. J.; Wang, B.; Ziegler, J. I.; Haglund, R. F.; Pantelides, S. T.; Bolotin, K. I. Bandgap Engineering of Strained Monolayer and Bilayer MoS_2. *Nano Lett.* 2013, 13 (8), 3626–3630. https://doi.org/10.1021/nl4014748.

(3) Xiao, J.; Zhang, Y.; Chen, H.; Xu, N.; Deng, S. Enhanced Performance of a Monolayer MoS_2/WSe_2 Heterojunction as a Photoelectrochemical Cathode. *Nanomicro Lett.* 2018, 10 (4), 60. https://doi.org/10.1007/s40820-018-0212-6.

(4) Latini, S.; Winther, K. T.; Olsen, T.; Thygesen, K. S. Interlayer Excitons and Band Alignment in MoS_2 / HBN / WSe_2 van Der Waals Heterostructures. *Nano Lett.* 2017, 17 (2), 938–945. https://doi.org/10.1021/acs.nanolett.6b04275.

(5) Chiu, M.-H.; Zhang, C.; Shiu, H.-W.; Chuu, C.-P.; Chen, C.-H.; Chang, C.-Y. S.; Chen, C.-H.; Chou, M.-Y.; Shih, C.-K.; Li, L.-J. Determination of Band Alignment in the Single-Layer MoS_2/WSe_2 Heterojunction. *Nat. Commun.* **2015**, 6 (1), 7666. https://doi.org/10.1038/ncomms8666.

(6) Tougaard, S. Practical Guide to the Use of Backgrounds in Quantitative XPS. *Journal of Vacuum Science & Technology A* **2021**, *39* (1). https://doi.org/10.1116/6.0000661.

(7) Tougaard, S. Improved XPS Analysis by Visual Inspection of the Survey Spectrum. *Surface and Interface Analysis* **2018**, *50* (6), 657–666. https://doi.org/10.1002/sia.6456.

(8) Shard, A. G. Practical Guides for X-Ray Photoelectron Spectroscopy: Quantitative XPS. *Journal of Vacuum Science & Technology A* **2020**, *38* (4). https://doi.org/10.1116/1.5141395.

(9) Xie, J.; Zhang, Z.; Zhang, H.; Nagarajan, V.; Zhao, W.; Kim, H.-L.; Sanborn, C.; Qi, R.; Chen, S.; Kahn, S.; Watanabe, K.; Taniguchi, T.; Zettl, A.; Crommie, M. F.; Analytis, J.; Wang, F. Low Resistance Contact to P-Type Monolayer WSe_2. *Nano Lett.* **2024**, 24 (20), 5937–5943. https://doi.org/10.1021/acs.nanolett.3c04195

(10) Fang, H.; Chuang, S.; Chang, T. C.; Takei, K.; Takahashi, T.; Javey, A. High-Performance Single Layered WSe_2 p-FETs with Chemically Doped Contacts. *Nano Lett.* **2012**, 12 (7), 3788–3792. https://doi.org/10.1021/nl301702r

(11) Chen, S.; Zhang, Y.; King, W. P.; Bashir, R.; van der Zande, A. M. Extension Doping with Low-Resistance Contacts for P-Type Monolayer WSe_2 Field-Effect Transistors. *Adv. Electron. Mater.* **2024**. https://doi.org/10.1002/aelm.202400843.

(12) Ho, P.-H.; Yang, Y.-Y.; Chou, S.-A.; Cheng, R.-H.; Pao, P.-H.; Cheng, C.-C.; Radu, I.; Chien, C.-H. High-Performance WSe_2 Top-Gate Devices with Strong Spacer Doping. *Nano Lett.* **2023**, 23 (22), 10236–10242. https://doi.org/10.1021/acs.nanolett.3co2757.

(13) Chiang, C.-C.; Lan, H.-Y.; Pang, C.-S.; Appenzeller, J.; Chen, Z. Air-Stable P-Doping in Record High-Performance Monolayer WSe_2 Devices. *IEEE Electron Device Letters* **2022**, 43 (2), 319–322. https://doi.org/10.1109/LED.2021.3135312.

(14) Kim, I.; Higashitarumizu, N.; Rahman, I. K. M. R.; Wang, S.; Kim, H. M.; Geng, J.; Prabhakar, R. R.; Ager, J. W.; Javey, A. Low Contact Resistance WSe_2 p-

Type Transistors with Highly Stable, CMOS-Compatible Dopants. *Nano Lett.* **2024**, 24 (43), 13528–13533. https://doi.org/10.1021/acs.nanolett.4c02948.

(15) Lan, H.-Y.; Tripathi, R.; Liu, X.; Appenzeller, J.; Chen, Z. Wafer-Scale CVD Monolayer WSe_2 p-FETs with Record-High 727 μA/μm Ion and 490 μS/μm gmax via Hybrid Charge Transfer and Molecular Doping. In *2023 International Electron Devices Meeting (IEDM)*; IEEE, **2023**; pp 1–4. https://doi.org/10.1109/IEDM45741.2023.10413736.

(16) Cai, L.; McClellan, C. J.; Koh, A. L.; Li, H.; Yalon, E.; Pop, E.; Zheng, X. Rapid Flame Synthesis of Atomically Thin MoO_3 down to Monolayer Thickness for Effective Hole Doping of WSe_2. *Nano Lett.* **2017**, *17* (6), 3854–3861. https://doi.org/10.1021/acs.nanolett.7b01322.

(17) Kwon, G.; Choi, Y.-H.; Lee, H.; Kim, H.-S.; Jeong, J.; Jeong, K.; Baik, M.; Kwon, H.; Ahn, J.; Lee, E.; Cho, M.-H. Interaction- and Defect-Free van Der Waals Contacts between Metals and Two-Dimensional Semiconductors. *Nat. Electron.* **2022**, *5* (4), 241–247. https://doi.org/10.1038/s41928-022-00746-6.

(18) Chou, A.-S.; Lin, Y.-T.; Lin, Y. C.; Hsu, C.-H.; Li, M.-Y.; Liew, S.-L.; Chou, S.-A.; Chen, H.-Y.; Chiu, H.-Y.; Ho, P.-H.; Hsu, M.-C.; Hsu, Y.-W.; Yang, N.;

Woon, W.-Y.; Liao, S.; Hou, D.-H.; Chien, C.-H.; Chang, W.-H.; Radu, I.; Wu, C.-I.; Philip Wong, H.-S.; Wang, H. High-Performance Monolayer WSe_2 p/n FETs via Antimony-Platinum Modulated Contact Technology towards 2D CMOS Electronics. In *2022 International Electron Devices Meeting (IEDM)*; IEEE, 2022; pp 7.2.1-7.2.4. https://doi.org/10.1109/IEDM45625.2022.10019491.

(19) Li, M.; Zhang, X.; Zhang, Z.; Peng, G.; Zhu, Z.; Li, J.; Qin, S.; Zhu, M. Unipolar P-Type Monolayer WSe_2 Field-Effect Transistors with High Current Density and Low Contact Resistance Enabled by van Der Waals Contacts. *Nano Research* 2024, *17* (11), 10162–10169. https://doi.org/10.1007/s12274-024-6942-5.
